# Platelet-to-hemoglobin ratio as a valuable predictor of long-term all-cause mortality in coronary artery disease patients with congestive heart failure

**DOI:** 10.1186/s12872-021-02423-6

**Published:** 2021-12-28

**Authors:** Kunming Bao, Haozhang Huang, Guoyong Huang, Junjie Wang, Ying Liao, Yuxiong Pan, Weihua Chen, Jin Lu, Yanfang Yang, Zhidong Huang, Shiqun Chen, Kaihong Chen, Liling Chen

**Affiliations:** 1 Department of Cardiology, Longyan First Affiliated Hospital of Fujian Medical University, Longyan, 364000 China; 2 Department of Cardiology, Guangdong Provincial Key Laboratory of Coronary Heart Disease Prevention, Guangdong Cardiovascular Institute, Guangdong Provincial People’s Hospital, Guangdong Academy of Medical Sciences, Guangzhou, 510080 China; 3 The Second School of Clinical Medicine, Southern Medical University, Guangzhou, 510515 China

**Keywords:** Coronary artery disease, Congestive heart failure, Mortality, Platelet-to-hemoglobin ratio

## Abstract

**Background:**

The platelet-to-hemoglobin ratio (PHR) has emerged as a prognostic biomarker in coronary artery disease (CAD) patients after PCI but not clear in CAD complicated with congestive heart failure (CHF). Hence, we aimed to assess the association between PHR and long-term all-cause mortality among CAD patients with CHF.

**Methods:**

Based on the registry at Guangdong Provincial People’s Hospital in China, we analyzed data of 2599 hospitalized patients who underwent coronary angiography (CAG) and were diagnosed with CAD complicated by CHF from January 2007 to December 2018. Low PHR was defined as ˂ 1.69 (group 1) and high PHR as ≥ 1.69 (group 2). Prognosis analysis was performed using Kaplan–Meier method. To assess the association between PHR and long-term all-cause mortality, a Cox-regression model was fitted.

**Results:**

During a median follow-up of 5.2 (3.1–7.8) years, a total of 985 (37.9%) patients died. On the Kaplan–Meier analysis, patients in high PHR group had a worse prognosis than those in low PHR group (log-rank, *p* = 0.0011). After adjustment for confounders, high PHR was correlated with an increased risk of long-term all-cause mortality in CAD patients complicated with CHF. (adjusted hazard ratio [aHR], 1.31; 95% confidence interval [CI], 1.13–1.52, *p* < 0.0001).

**Conclusion:**

Elevated PHR is correlated with an increased risk of long-term all-cause mortality in CAD patients with CHF. These results indicate that PHR may be a useful prognostic biomarker for this population. Meanwhile, it is necessary to take effective preventive measures to regulate both hemoglobin levels and platelet counts in this population.

**Supplementary Information:**

The online version contains supplementary material available at 10.1186/s12872-021-02423-6.

## Introduction

Coronary artery disease (CAD) is the leading cause of morbidity and mortality globally. CAD complicated by heart failure especially carries considerable morbidity and poor prognosis [1]. Those facts indicate that it is necessary to quest useful and simple indicators to evaluate the prognosis of CAD patients complicated with congestive heart failure (CHF) for effective and timely intervention strategies.

A growing body of literature has illustrated the prognostic utility of various complete blood counts in predicting adverse outcomes in cardiovascular disease [2, 3]. High circulating platelet counts have been reported to be associated with poor outcomes in cardiovascular disease [4–6], the mechanism of which may be inflammatory response [7, 8] and platelet activation [9]. In contrast, low hemoglobin levels were considered as poor prognostic factors of cardiovascular disease [10, 11], owing to worsening myocardial ischemia [12], neurohormonal activation, increased cardiac output [13, 14] and adverse left ventricular (LV) remodeling [14, 15].

Platelet-to-hemoglobin ratio has emerged as a novel and readily available prognostic parameter in cardiovascular disease. Zheng et al. [16] reported that PHR was an independent predictor of adverse outcomes in CAD patients who underwent percutaneous coronary intervention (PCI) and was considered as a stronger predictor than platelet counts or hemoglobin levels alone. However, the association of PHR with all-cause mortality in CAD patients with congestive heart failure is not clear. In this context, we aimed to investigate the relationship between PHR and long-term all-cause mortality of CAD patients with CHF.

## Population and methods

### Data sources and study population

**Fig. 1:**
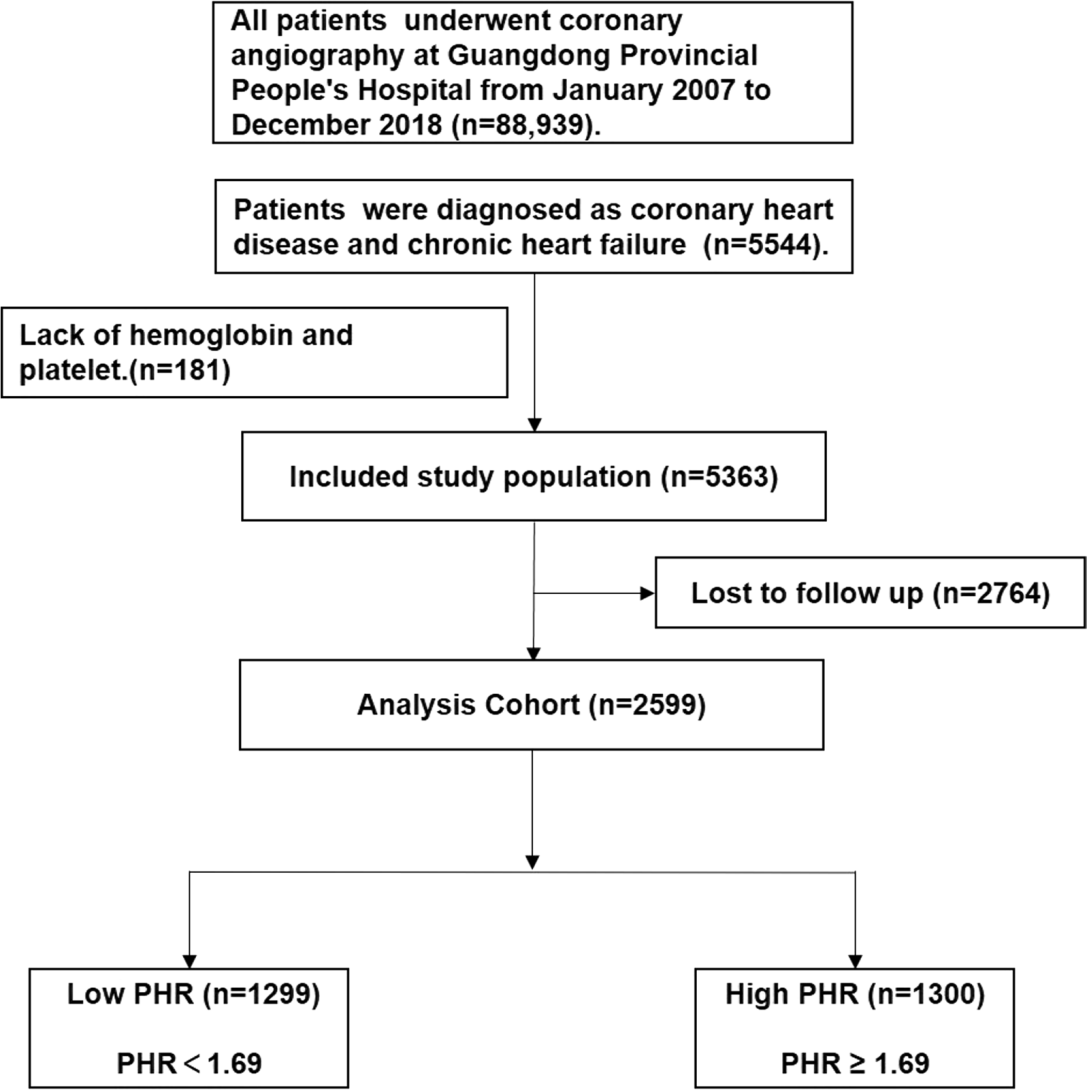
Patients flow diagram

This is an observational cohort, single-center and retrospective study. The data we used in this study was based on the electronic clinical management records system of the Guangdong Provincial People’s Hospital (ClinicalTrials.gov NCT04407936). We collected data on all-cause mortality through the Guangdong Provincial Public Security and then matched to the electronic clinical management system of the Guangdong Provincial People’s Hospital records. The baseline data included demographic characteristics, medical history, laboratory test results and medication use. We included patients undergoing coronary angiography (CAG) and with a final diagnosis of CAD complicated by CHF in accordance with the 10th Revision Codes of the International Classification of Diseases (ICD-10; I20.xx–I25.xx, I50.00001, and I91.40001, Additional file 1: Table S1) from January 2007 to December 2018. Percutaneous coronary intervention (PCI) or coronary angiography (CAG) was performed in accordance with authoritative clinical practice guidelines [17, 18]. All blood samples were collected in the early morning after overnight fasting. We excluded patients who lacked platelet counts, hemoglobin levels and follow-up information. Finally, 2599 patients were enrolled in this analysis (Fig. 1). The study population was dichotomized based on the PHR on admission according to the median. We defined PHR ˂ 1.69 as low PHR (group 1), PHR ≥ 1.69 as high PHR (group 2).

### Clinical definition

CHF was defined as New York Heart Association (NYHA) class > 2 or Killip class > 1 [19]. Acute myocardial infarction (AMI) was defined as having a medical history of an ST-elevated myocardial infarction (STEMI) or Non-STEMI cardiac events. Chronic kidney disease (CKD) was defined as an estimated glomerular filtration rate (eGFR) < 60 mL/min/1.73 m^2^. eGFR was calculated according to the Modification of Diet in Renal Disease (MDRD) equation. Hypertension (HT) and diabetes mellitus (DM) were defined following the 10th Revision Codes of the International Classification of Diseases.

### Study endpoints and clinical follow-up

Long-term all-cause mortality was the primary endpoint of this study which was defined as any death recorded from the date of enrollment to the date of the last follow-up visit. Follow-up time and data on long-term all-cause mortality were obtained from the Guangdong Provincial Public Security and then matched to the electronic Clinical Management System of the Guangdong Provincial People’s Hospital records.

### Statistical analysis

Descriptive statistics on baseline variables are presented as the mean (standard deviation [SD]), median (interquartile range [IQR]), or number and percentage as appropriate. Differences in baseline characteristics between groups were analyzed by Student’s t-test when appropriate. The categorical data was analyzed by Pearson chi-squared tests. Restricted cubic splines were used to investigate the associations of PHR with long-term all-cause death. Survival times were plotted using Kaplan–Meier survival curves, the log-rank test was used to compare differences in survival.

The usefulness of PHR for independently predicting long-term all-cause mortality among CAD patients with CHF was analyzed by the Cox regression models. Hazard ratios (HRs) and 95% confidence intervals (CIs) are reported. Those related to mortality on the basis of clinical experience were further controlled using multivariable Cox regression in 3 different models. Model 1 was unadjusted, model 2 was adjusted for age and gender, model 3 included model 2 variables, medical history (CKD, HT, AMI, Stroke, DM, pre-acute myocardial infarction and PCI) and drugs information (angiotensin-converting enzyme inhibitor/angiotensin receptor blockers, aspirin, β-blockers, clopidogrel and statins). We conducted a sensitivity analysis with categorization to quartiles groups (group 1 (0.02–1.34), group 2 (1.34–1.69), group 3 (1.69–2.15), group 4 (2.15–9.32)) in order to evaluate the trend of the association and whether there is a gradual, stepwise association. Subgroup analysis was performed among 8 prespecified subgroups (age ≥ 75 or age < 75, male or female, non-CKD or CKD, non-PCI or PCI) to assess the association of PHR with long-term all-cause mortality among CAD patients with CHF.

All P values were calculated with two-sided tests. A threshold of *p*-value < 0.05 was set to represent statistical significance. All data analyses were performed by R software (version 4.0.3; R Core Team, Vienna, Austria).

## Results

### Clinical characteristics

A total of 2599 patients were included in the study. The baseline clinical characteristics of the patients are shown in Table 1. Among the whole study population, the mean age was 66.3 ± 10.9 years, and 660 (25.4%) were female. A total of 1857 (71.5%) patients underwent PCI treatment, 1290 (49.7%) patients were diagnosed as AMI, 1117 (43.0%) patients were identified as having CKD, 921 (35.5%) patients had DM. Patients in high PHR group were more likely to be female (31.3%) which were positively associated with the prevalence of AMI, DM, HT, CKD and negatively associated with admission ALB, eGFR, HGB levels. There was significantly higher use of statins, clopidogrel, aspirin and calcium channel blockers in the high PHR group. More details of the baseline characteristics of patients enrolled are shown in Table 1.

**Table 1:** Baseline characteristics of the study groups

Characteristic	Overall	Group1	Group2	*P* value
	(n = 2599)	PHR < 1.69	PHR ≥ 1.69	
		(n = 1299)	(n = 1300)	
*Demographic characteristics*				
Age, years, mean (SD)	66.26 (10.91)	66.29 (10.76)	66.24 (11.07)	0.907
Age ≥ 75 (%)	651 (25.0)	334 (25.7)	317 (24.4)	0.462
Female, n (%)	660 (25.4)	253 (19.5)	407 (31.3)	< 0.001
*Medical history*				
DM, n (%)	921 (35.5)	405 (31.2)	516 (39.7)	< 0.001
AMI, n (%)	1290 (49.7)	592 (45.6)	698 (53.7)	< 0.001
HT, n (%)	1504 (57.9)	698 (53.8)	806 (62.0)	< 0.001
CKD, n (%)	1117 (43.0)	523 (40.3)	594 (45.7)	0.006
Hyperlipidemia, n (%)	1726 (69.5)	855 (69.1)	871 (69.9)	0.703
LVEF mean (SD)	48.55 (14.50)	48.57 (14.94)	48.53 (14.06)	0.957
Anemia, n (%)	1319 (50.8)	471 (36.3)	848 (65.2)	< 0.001
Stroke, n (%)	213 (8.2)	108 (8.3)	105 (8.1)	0.877
PCI, n (%)	1857 (71.5)	904 (69.6)	953 (73.3)	0.040
*Laboratory tests*				
WBC, 109/L, mean (SD)	9.57 (4.08)	9.25 (4.05)	9.88 (4.08)	< 0.001
HGB, g/L, mean (SD)	126.07 (20.42)	134.13 (17.51)	118.03 (19.94)	< 0.001
PLT, 109/L, mean (SD)	227.00 (85.61)	172.66 (39.05)	281.29 (85.03)	< 0.001
ALB, g/L, mean (SD)	33.09 (4.87)	33.81 (4.73)	32.36 (4.92)	< 0.001
eGFR, ml/min/1.73 m^2^, mean (SD)	62.87 (28.12)	65.40 (25.60)	60.31 (30.25)	< 0.001
HbA1c, %, mean (SD)	6.82 (1.60)	6.70 (1.48)	6.95 (1.69)	0.003
CHOL, mmol/L, mean (SD)	4.52 (1.25)	4.52 (1.21)	4.52 (1.28)	0.983
TRIG, mmol/L, mean (SD)	1.50 (0.92)	1.46 (0.98)	1.53 (0.86)	0.047
HDL-C, mmol/L, mean (SD)	0.97 (0.27)	0.98 (0.27)	0.96 (0.27)	0.125
LDL-C, mmol/L, mean (SD)	2.83 (1.03)	2.84 (1.01)	2.82 (1.04)	0.562
*Medications*				
Beta-blocker, n (%)	1781 (77.6)	903 (77.7)	878 (77.5)	0.940
ACEI/ARB, n (%)	1137 (49.5)	596 (51.3)	541 (47.7)	0.098
Clopidogrel, n (%)	1954 (85.1)	956 (82.3)	998 (88.1)	< 0.001
Aspirin, n (%)	1987 (86.6)	983 (84.6)	1004 (88.6)	0.006
Statins, n (%)	2069 (90.2)	1025 (88.2)	1044 (92.1)	0.002
CCB, n (%)	437 (19.0)	190 (16.4)	247 (21.8)	0.001

DM, diabetes mellitus; AMI, acute myocardial infarction; HT, hypertension; CKD, chronic kidney disease; LVEF, left ventricular ejection fraction; PCI, percutaneous coronary intervention; WBC, white blood cell; HGB, hemoglobin; PLT, platelet; ALB: albumin; eGFR, estimated glomerular filtration rate; HbA1c, hemoglobin A1c; CHOL, cholesterol; TRIG, triglycerides; HDL-C, high-density lipoprotein cholesterol; LDL-C, low-density lipoprotein cholesterol; ACEI/ARB, angiotensin-converting enzyme inhibitor/angiotensin receptor blocker; CCB, calcium channel blockers

### Primary outcomes

**Fig. 2:**
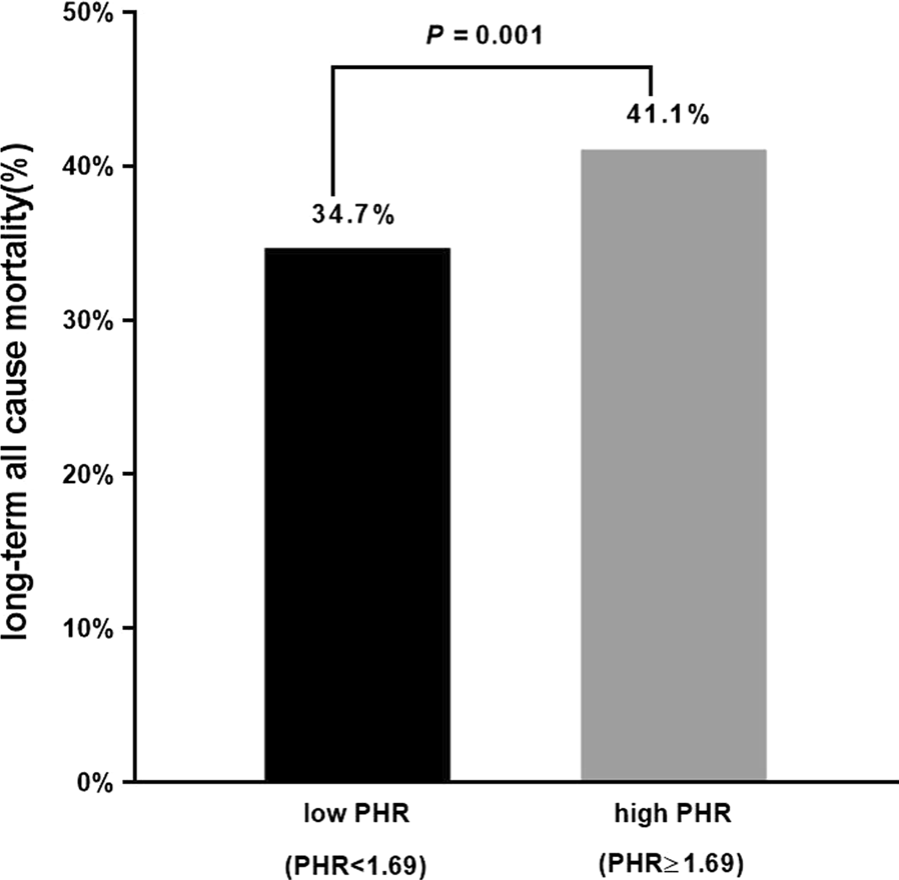
Bar chart for long-term all-cause mortality of PHR

**Fig. 3:**
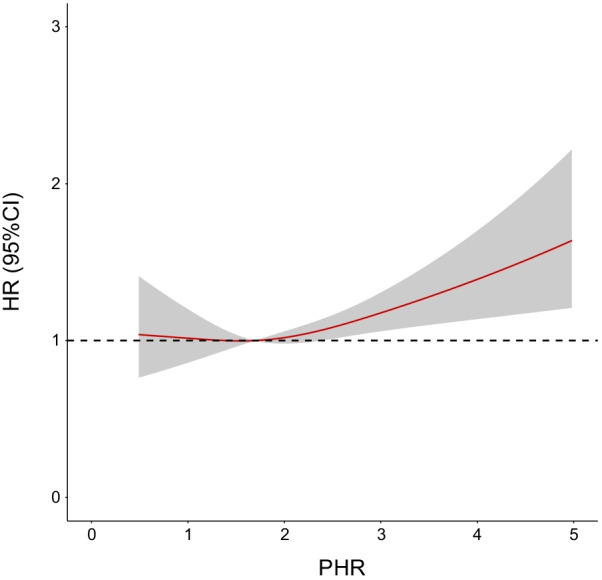
Restricted cubic spline curve for the PHR hazard ratio

**Fig. 4:**
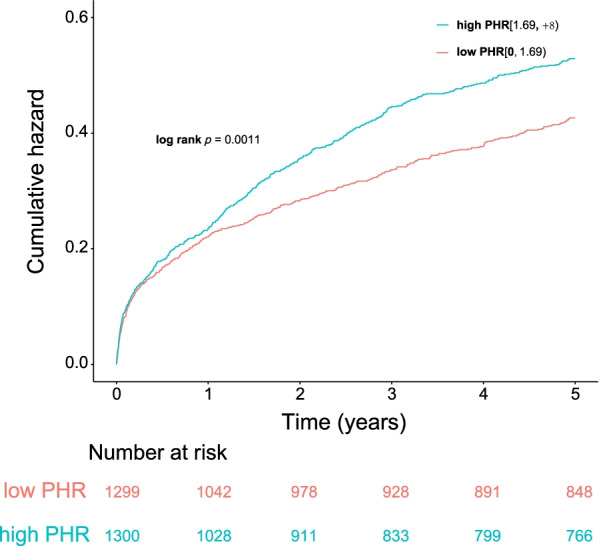
Kaplan–Meier curves for long-term all-cause mortality of PHR

During a median follow-up of 5.2 (3.1–7.8) years, a total of 985 (37.9%) patients died (*p* = 0.001; Fig. 2). Restricted cubic splines showed that HR for the primary endpoint was positively associated with PHR, but the relationship between them was nonlinear (Fig. 3). As determined by Kaplan–Meier analysis, the high PHR group had a higher incidence of long-term all-cause mortality, the statistically significant differences between KM curves were measured by the log-rank test (log-rank, *p* = 0.0011; Fig. 4).

**Fig. 5:**
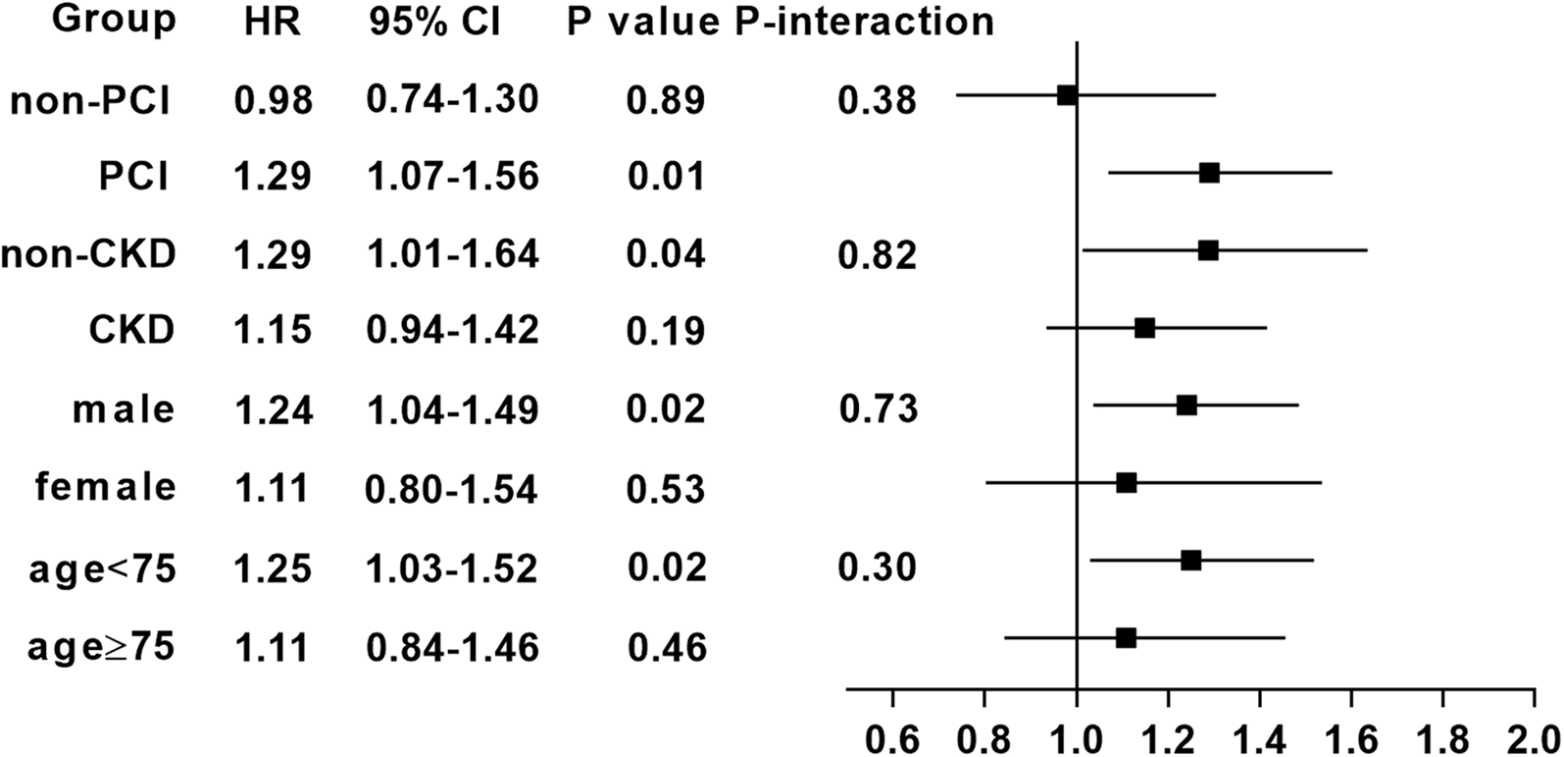
Forest plots of hazard ratios for the primary endpoint in different subgroups

The relationship between high PHR and long-term all-cause mortality was evaluated using Cox proportional hazards models. Our results demonstrated that high PHR was associated with a higher risk of all-cause death than low PHR even after full adjustment for major confounders. (model 1: HR 1.23, 95% CI 1.09–1.40, *p* < 0.0001; model 2: HR 1.26, 95% CI 1.11–1.43, *p* < 0.0001; model 3: HR 1.31, 95% CI 1.13–1.52, *p* < 0.0001; Table 2). Increasing over 30% higher risk among CAD with CHF in the highest quartile compared to the lowest (HR 1.36, 95% CI 1.11–1.66; Table 3). At the same time, a gradual, stepwise association was observed (P for trend < 0.05). In a subgroup analysis, the Cox regression analysis revealed that high PHR had a consistently higher relative risk of mortality among age < 75, male, non-CKD and PCI subgroups (Fig. 5).

**Table 2:** Cox proportional hazards model for the association between PHR and long-term all-cause mortality

Groups	N	HR, 95% CI, *p* value		
		Model 1*	Model 2^$^	Model 3^§^
Low PHR	1299	Ref	Ref	Ref
High PHR	1300	1.23 (1.09–1.40), < 0.0001	1.26 (1.11–1.43), < 0.0001	1.31 (1.13–1.52), < 0.0001

^*^Unadjusted

^$^Adjusted for age and gender

^§^Adjusted for full multivariate: age, gender, chronic kidney disease, hypertension, acute myocardial infarction, pre-acute myocardial infarction, stroke, diabetes mellitus, percutaneous coronary intervention, angiotensin-converting enzyme inhibitor/angiotensin receptor blockers, β-blockers, aspirin, statins and clopidogrel

**Table 3:** Sensitivity analysis for relationship of PHR and long-term all-cause mortality with categorization to quartiles groups

Quartiles (min–max)	HR, 95% CI, *p* value
Group 1 (0.02–1.34)	Ref
Group 2 (1.34–1.69)	0.86 (0.69–1.06), *p* = 0.16
Group 3 (1.69–2.15)	1.07 (0.86–1.32), *p* = 0.54
Group 4 (2.15–9.32)	1.36 (1.11–1.66), *p* < 0.0001
*p* for trend	< 0.05

Adjusted for full multivariate: age, gender, chronic kidney disease, hypertension, acute myocardial infarction, pre-acute myocardial infarction, stroke, diabetes mellitus, percutaneous coronary intervention, angiotensin-converting enzyme inhibitor/angiotensin receptor blockers, β-blockers, aspirin, statins and clopidogrel

## Discussion

This study demonstrated the association of PHR with all-cause mortality among CAD patients with CHF. Our study showed that long-term all-cause mortality of this population was approximately 40% in a median follow-up of 5.2 years and high PHR increased long-term all-cause mortality by 31% after adjusting major confounders among those patients.

CAD is often complicated by heart failure, which leads to a worse prognosis [20, 21]. Some scholars found that about 20–30% of long-term all-cause mortality in a median follow-up of ~ 2 years [22, 23], which were lower than our data. It suggested that more information about endpoints may be attained during a longer follow-up time. CAD is a common reason for HF development [24]. Either AMI or chronic ischemia leads to LV remodeling, ischemic mitral regurgitation and LV dysfunction [25, 26]. In acute heart failure syndromes, the high LV diastolic pressure and low systemic blood pressure often result in subendocardial ischemia and lead to worse outcomes [27]. CAD patients complicated by LV dysfunction carry a high risk for sudden cardiac death because of recurrent myocardial injury or the abrupt onset of ventricular arrhythmias [28, 29]. Thus, physicians need some simple indicators to evaluate the outcomes among CAD patients complicated with CHF.

The predictive value of other blood parameters, such as lymphocytes, monocytes and high-density lipoprotein cholesterol, for clinical outcomes has been demonstrated in the field of cardiovascular disease [30]. Neutrophil-to-lymphocyte ratio (NLR) and platelet-to-lymphocyte ratio (PLR) have been reported to be useful markers to predict poor prognosis in CAD patients [2, 31]. It is acknowledged that CAD patients with CHF are at high risk for adverse outcomes. Recently, platelet-to-hemoglobin ratio has emerged as a novel prognostic predictor for cardiovascular disease. Zheng et al. [16] showed that PHR was an independent prognostic marker for CAD patients after PCI with better prognostic value than absolute platelet counts or hemoglobin levels. Nevertheless, evidence of the prognostic value of PHR for CAD patients with CHF is lacking. As a complement, our study indicated that PHR was significantly associated with poor outcomes among CAD patients with CHF.

The role of PHR in the deterioration of CAD with CHF remains unclear. On one hand, persistent inflammation is a hallmark of coronary artery disease and congestive heart failure [7, 8]. The release of various mediators during a proinflammatory state results in megakaryocyte proliferation and increased platelet counts in circulation [9, 32, 33], which may indicate elevated platelet activation and a prothrombotic state [34, 35]. These processes may cause thrombosis-related complications in CHF patients [4, 5]. In terms of CAD, activated platelets play a vital role in the development and progression of atherosclerosis [36], the instability of atherosclerotic plaques and thrombus formation in the case of vascular endothelium injury and plaque rupture [32, 37, 38]. On the other hand, low hemoglobin levels indicate decreased oxygen-carrying capacity, which may worsen the myocardial ischemic injury [12]. Subsequent tissue hypoxia may result in the activation of sympathetic nervous system and renin–angiotensin–aldosterone systems, which eventually result in elevated cardiac output [13, 14]. These changes may chronically lead to adverse LV remodelling [14, 15] and a vicious cycle of HF progression [25].

In addition, there is a potential mechanistic association between reduced hemoglobin and elevated platelet count. Firstly, anemia can increase whole blood aggregometry, artifactually promoting platelet aggregation. Then, the low hemoglobin often coexists with inflammation. Inflammatory biomarkers, such as fibrinogen, von Willebrand factor, and inflammatory cytokines may directly increase platelet reactivity. Moreover, due to the anemic milieu, bone marrow become hyperactivity and release more platelets subsequently [39].

Complete blood count is performed routinely upon admission and frequently repeated during hospitalization in all CAD patients. PHR is an easily calculated, readily available and reproducible biomarker with no further cost for the patient or healthcare system. CAD complicated by CHF carries considerable morbidity and poor prognosis. It is necessary to take some measures to improve the prognosis of this population, such as secondary prevention of CAD and appropriate management of congestive heart failure. As to high PHR patients, controlling the progression of inflammatory response, the administration of antiplatelet or anticoagulant medications and supplement of erythrotropin analogs or iron for improvement of anemia are essential and beneficial to postpone ventricular remodeling and improve prognosis.

## Limitations

This study examined the significance of PHR on long-term all-cause death among CAD patients with CHF. There are several limitations of this study. First, information about cause-specific death was not available in this study, which restricted our ability to examine the significance of PHR with cause-specific death, such as cardiovascular disease mortality. Second, only on admission baseline platelet counts and hemoglobin levels were contained in our study. Therefore, we could not know the variation trends of platelet counts and hemoglobin levels during hospitalization and the effects of such changes. Third, although we have adjusted for many confounders in the analysis, there could be some residual confounding due to unmeasured factors. Fourth, in this study, we did not exclude patients with chronic liver disease, chronic lung disease…, which may influence hemoglobin and platelet levels.

## Conclusions

High PHR is a novel, independent predictor of long-term all-cause mortality in CAD patients with CHF. It is helpful for risk stratification in CAD patients complicated by CHF to identify high-risk patients for further targeted intervention. However, prospective multi-center cohort studies are required to provide high-level of evidence and validate our findings.

## Supplementary Information

**Additional file 1: Table S1**. The ICD-10 codes information of diagnosis

## Data Availability

The datasets used and/or analyzed during the current study are available from the corresponding author on reasonable request.

## Abbreviations

PHR: Platelet-to-hemoglobin ratio
CAD: Coronary artery disease
CHF: Congestive heart failure
CAG: Coronary angiography
PCI: Percutaneous coronary intervention
LV: Left ventricular
NYHA: New York Heart Association
CKD: Chronic kidney disease
eGFR: Estimated glomerular filtration rate
MDRD: Modification of diet in renal disease
AMI: Acute myocardial infarction
STEMI: ST-elevated myocardial infarction
DM: Diabetes mellitus
HT: Hypertension
